# Abbreviated emergency laparotomy in the non-trauma setting

**DOI:** 10.1186/1749-7922-4-41

**Published:** 2009-11-19

**Authors:** Benjamin Person, Tatiana Dorfman, Hany Bahouth, Amira Osman, Ahmad Assalia, Yoram Kluger

**Affiliations:** 1 Department of General Surgery B, Rambam Health Care Campus, POB 9602, Haifa, 31096 Israel

## Abstract

**Background:**

Although the application of damage control surgery for trauma has been widely reported and defined, similar approach in non-trauma patients has not been well detailed.

**Methods:**

A retrospective analysis of data from non-trauma patients who underwent emergency laparotomy between May 2006 and December 2008. Demographics, indications for surgery and outcome of patients who had definitive laparotomies (DL) and patients who had abbreviated laparotomies (AL) were compared. Appendectomies were excluded.

**Results and discussion:**

Two-hundred ninety-one patients (55% males) were included. Thirty-one (10.7%) underwent AL (58% males). Mean age of patients who had DL and AL was 65 and 62.8 years respectively. Peritonitis and mesenteric ischemia were more common indications in patients with AL than DL: 48.4% vs. 30.4% (p = 0.04) and 32.3% vs. 3.5% (p < 0.0001) respectively. Only 29% of patients who had AL were hemodynamically unstable. Mortality rates were 54.8% and 16.5% in patients with AL and DL respectively (p < 0.0001). Patients who died after AL and DL were significantly older than patients who survived (75 vs. 47.3 and 74 vs. 63 years respectively, p < 0.0001). Median hospital stay was 21 and 9 days for patients with AL and DL respectively (p < 0.05). Patients who underwent AL had significantly more wound infections, sepsis and multi-organ failure.

**Conclusion:**

The philosophy of damage control surgery is applied to non-trauma patients as some of the prerequisites for the decision to elect this strategy are the same. Peritonitis is the most common indication for AL in non-trauma patients.

## Background

Since the earliest descriptions of intentionally abbreviated laparotomy more than 20 years ago [1-3], damage-control laparotomy has been widely applied in severely traumatized patients and extensively scrutinized in the literature. The realization that correction of metabolic failure rather than anatomic perfection is mandatory for immediate survival led to the development of this approach. The "lethal triad" of hypothermia, acidosis, and coagulopathy was viewed as a vicious cycle that often could not be interrupted and which marked the limit of the patient's ability to cope with the physiological consequences of injury, at which point prolongation of the operation frequently resulted in the patient's demise. The principles and sequence of damage control include an abbreviated laparotomy for control of massive bleeding and fecal spillage, secondary correction of abnormal physiological parameters in an intensive care setting followed by a planned definitive re-exploration for correction of anatomical derangements [4,5]. Even though that overall morbidity remained relatively high, the application of this technique proved beneficial in reducing mortality rates and improving survival.

In many instances, the acute-care surgeon is faced with non-trauma patients in whom the philosophy of damage control surgery and especially early abbreviation of the index surgery may be appealing and well appropriate. Metabolic disturbances (acidosis), peritonitis and peritoneal fecal load as well as hemodynamic instability are commonly encountered in a wide variety of disease processes. The concept of abbreviated surgery in non-trauma patients is rarely discussed in the literature [6-11]. The indications for abbreviation of emergency laparotomy in the non-trauma setting as well as patients' characteristics and outcomes are not well-defined. In this article we report our experience with abbreviated laparotomy surgery in non-trauma patients.

## Methods

The objectives of the current study were to delineate the indications and reasons for abbreviated surgery decided upon by senior surgeons in the department of surgery in our institution and to assess the outcome of non-trauma patients who underwent emergency laparotomy for acute abdominal processes. This aim was achieved by conducting a retrospective data analysis of the medical records of all the patients 17 years of age and older who underwent an emergency laparotomy in a non-trauma setting between May 2006 and December 2008 in our department. Patients in whom the diagnosis was appendicitis were excluded. Two groups of patients were compared: patients who underwent an abbreviated laparotomy (AL), and patients who had a definitive laparotomy (DL). Analyzed parameters included demographics, indications for emergency surgery, number of laparotomies performed in each group (planned and unplanned), length of hospital stay (LOS), morbidity and mortality. Hemodynamic instability was defined as a systolic blood pressure lower than 100 mmHg and a heart rate higher than 100 on admissions to the emergency department. Statistical analysis was performed using the Fisher's Exact Test; significant differences were determined when p was smaller than 0.05.

## Results

The medical records of 291 patients (55% males) who underwent an emergency laparotomy during the study period were analyzed. Thirty-one patients (10.7%) underwent AL (58% males). Mean age of patients who had DL and AL was 65.0 (19-96) and 62.8 (25-96) years respectively. Peritonitis and mesenteric ischemia were significantly more common indications for emergency laparotomy in patients who underwent AL than patients who underwent DL: 48.4% vs. 30.4% (p = 0.04) and 32.3% vs. 3.5% (p < 0.0001) respectively; whereas intestinal obstruction was significantly more common in patients who had DL compared to those who had AL: 58.1% vs. 6.5% (p < 0.0001). Intra-abdominal/gastrointestinal bleeding comprised 9.7% of patients who had AL and 3.1% of patients who had DL (p = NS). Emergency laparotomy for all other indications was performed in one patient (3.2%) in the AL group and in 13 patients (5%) in the DL group (p = NS) (Table 1). Nine patients (29%) in the AL group were hemodynamically unstable on admission to the emergency department. All the patients in the DL group were stable on admission. The number and distribution of laparotomies in each group are summarized in Table 2. In the DL group, 19 patients (7.3%) had a second unplanned laparotomy, and 5 additional patients (1.9%) had 2 or more subsequent laparotomies following the first emergency operation (a total of at least 3 laparotomies). A total of 24 patients in the DL group (9.2%) underwent at least one unplanned laparotomy. Mortality rates were 54.8% and 16.5% in the AL and DL groups respectively (p < 0.0001). The most common cause of death in both groups was multi-organ failure (MOF) due to irreversible septic shock. In both groups the patients who died were significantly older than those who survived (75 vs. 47.3 years in the AL group and 74 vs. 63 years in the DL group; p < 0.0001 in each group), but there was no statistical difference between the two group with regard to the age of patients who died. Wound infection, MOF and sepsis [12] were significantly more frequent in patients in the AL group (Table 3). Median length of hospital stay (LOS) was significantly longer in the patients in the AL group (21 vs. 9 days; p < 0.05).

**Table 1:** Demographics and indications for emergency surgery

	AL	DL	p
**N patients (%)**	31 (10.7)	260 (89.3)	

**Male %**	58.1	54.2	NS

**Mean age (years)**	62.8 (± 18.8)	65.0 (± 17.7)	NS

**Peritonitis**	48.4%	30.4%	0.04

**Mesenteric ischemia**	32.3%	3.5%	< 0.0001

**Intestinal obstruction**	6.5%	58%	< 0.0001

**Bleeding**	9.7%	3.1%	NS

**Other**	3.2%	5.0%	NS

**Table 2:** Number of laparotomies in each group

	N -- Laparotomies			
	
	1	2	3+	Total
**AL - n (%)**	5 (16.1)	12 (38.7)	14 (45.2)	31 (100)

**DL -- n (%)**	236 (90.8)	19* (7.3)	5* (1.9)	260 (100)

**Total -- n (%)**	241 (82.8)	31 (10.7)	19 (6.5)	291 (100)

*- unplanned laparotomies

**Table 3:** Mortality and morbidity

	AL	DL	p
**Mortality**	54.8%	16.5%	< 0.0001

**Mean age:**	75 vs. 47.3	74 vs. 63.2	NS
**Died vs. survived**	P < 0.0001	P < 0.0001	

**Wound infection**	32.3%	13.3%	0.013

**MOF**	93.5%	21.5%	< 0.0001

**Sepsis**	83.9%	21.5%	< 0.0001

## Discussion

Damage control surgery made a monumental change in the paradigm that anatomical perfection must be achieved during the initial operation of critically injured patients. Trauma surgeons realized that the need to reverse the physiological "lethal triad" of acidosis, hypothermia and coagulopathy surpassed the necessity to correct all the anatomical derangements that were caused by the initial injury. Definitive surgery in the acute setting is practiced under strict adherence to a pre-defined algorithm in which damage control surgery is elected for the most seriously injured, and some of the indications for damage control in trauma may be applied for non-trauma critically ill patients as well.

There is little level I evidence to support abbreviated surgery in a non-trauma setting. In the only randomized, non-blinded, multi-center clinical trial, 232 patients who had severe secondary peritonitis were randomly assigned to undergo either a planned re-laparotomy or an "on-demand" laparotomy - 116 patients in each group [13]. The primary end point was death and/or peritonitis-related morbidity within a 12-month follow-up period. Secondary end points included health care utilization and costs. There were no significant differences in the primary end points between the two groups. A total of 42% of the patients in the "on-demand" group had a re-laparotomy vs. 94% of the patients in the planned re-laparotomy group. A total of 31% of first re-laparotomies were nontherapeutic in the "on-demand" group vs. 66% in the planned re-laparotomy group (p < 0.001), a finding that is not encouraging in support of a strategy of planned re-laparotomy. Patients in the "on-demand" group had shorter median intensive care unit stays (7 vs. 11 days; p = 0.001) and shorter median hospital stays (27 vs. 35 days; p = 0.008). Direct medical costs per patient were reduced by 23% using the on-demand strategy. The conclusions of this study were that on demand rather than planned re-laparotomy may therefore be considered the preferred surgical strategy in patients with severe peritonitis. This multi-center randomized trial focused on patients with secondary peritonitis due to conditions such as gastrointestinal perforation, mesenteric ischemia and anastamotic leakage, with systemic manifestations of sepsis. Of note, patients with pancreatitis and patients requiring "damage-control" procedures with mandatory re-explorations (e.g., abdominal packs left in, stapled bowel ends left in) were excluded from the study. Therefore, these results may not be applied to the sickest patients - those with so much contamination, necrosis, edema or physiologic instability that abbreviation of the index operation, repeat laparotomy and delayed closure were deemed imperative by the surgical team. These patients, who might arguably be the greatest beneficiaries of a planned re-laparotomy approach, were excluded from the study. Despite the decisive results in favor of on-demand re-laparotomy, there still appears to be a role, maybe even a necessity, for planned re-laparotomy as an exit strategy in selected unstable patients. These patients were the main focus of our study, a fact that accounts for the significant differences that we demonstrated between the AL and DL groups.

In an earlier multi-center, multi-national case-controlled trial [14], 38 patients who underwent planned re-laparotomy for the treatment of intra-abdominal infections were compared with 38 matched patients who had an on-demand re-laparotomy. A planned re-laparotomy was defined as at least one re-laparotomy decided on at the time of the first operation and the main outcome measures were morbidity and mortality. There was no significant difference in mortality between the two groups of patients, however, multi-organ failure and other infectious complications occurred significantly more frequently in the patients who underwent planned re-laparotomy. Although the authors of this paper concluded that those outcomes resulted from the operative strategy that was chosen, and recommended a cautious approach when evaluating the indications for planned re-laparotomy, we believe that these results actually emphasize the differences in the severity of the disease process between the two groups which led the surgical teams to choose a planned approach in the first place.

Lamme et al. conducted a meta-analysis of re-laparotomy for secondary peritonitis [15]. The analysis included 8 observational studies with a total of 1266 patients (286 in the planned re-laparotomy group and 980 in the re-laparotomy on demand group) and the primary outcome measure was in-hospital mortality. The combined results showed a statistically non-significant reduction in mortality for the on-demand re-laparotomy group compared with the planned re-laparotomy group of patients; however, due to the heterogeneity of the included studies, and the fact that none of them was randomized, the evidence generated by this meta-analysis was inconclusive.

In our department, 2 senior surgeons (HB and YK) are also fully trained in trauma and emergency surgery, which accounts for a generally increased awareness for concepts adapted from these fields, including that of damage control surgery. We found statistically significant differences between the DL and AL groups both in the rates of mortality and in the rates of significant morbidity; however, as mentioned earlier, we believe that these variations are due to differences in the severity of the disease processes between the two groups rather than the surgical approach that was selected. We also found that older age was a significant risk factor for mortality in both groups with significantly younger patients surviving both operative strategies.

The shortcomings of this report are that it is a retrospective analysis of data that are sometimes difficult to assess, and that we did not have all the parameters for objectively calculating the severity of the disease in each patient with a validated system such as the Acute Physiology and Chronic Health Evaluation II (APACHE II) score. A prospective, randomized trial may address these issues in a more precise manner.

## Conclusion

General surgeons encounter emergency abdominal catastrophes throughout their careers.

Innovation and unorthodox surgical practice are occasionally required for patients' salvage but such philosophy is not well defined in acute non-trauma settings.

Damage control strategies were proved to save lives among the injured. Applying similar principles to patients inflicted by abdominal surgical diseases with the same physiological derangements may prove beneficial as well.

## Competing interests

The authors declare that they have no competing interests.

## Authors' contributions

All authors read and approved the final manuscript. BP participated in the design of the study, performed the statistical analysis, drafted the manuscript and provided critical revisions of all versions of the manuscript. TD carried out the acquisition of the data and helped with the statistical analysis. HB provided critical revision of the manuscript. AO carried out the acquisition of the data and helped with the statistical analysis. AA provided critical revision of the manuscript. YK conceived of the study, and participated in its design and coordination and helped to draft the manuscript.
